# Supplementary material for: Transferring principles of solid-state and Laplace NMR to the field of in vivo brain MRI
Source: Magn Reson (Gott). 2020 Feb 28;1(1):27–43. doi: 10.5194/mr-1-27-2020 (PMC10500744; doi:10.5194/mr-1-27-2020)
Supplement: The supplement related to this article is available online at: https://doi.org/10.5194/mr-1-27-2020-supplement. [file mr-1-27-supplement.pdf]

Supplement of Magn. Reson., 1, 27–43, 2020
https://doi.org/10.5194/mr-1-27-2020-supplement
© Author(s) 2020. This work is distributed under
the Creative Commons Attribution 4.0 License.

Open Access
MAGNETIC RESONANCE

Supplement of

Transferring principles of solid-state and Laplace NMR to the field of in vivo brain MRI

João P. de Almeida Martins et al.

Correspondence to: João P. de Almeida Martins (joao.martins@fkem1.lu.se)

The copyright of individual parts of the supplement might differ from the CC BY 4.0 License.

15 MINUTES PROTOCOL

Two different 5D relaxation-diffusion MRI protocols were included in a single imaging session: the 45 min protocol described in the main manuscript, and a shortened 15 min protocol. The two protocols were consecutively used without repositioning the subject (female, 31 years). Here, we focus on the 15 min protocol whose main features are summarized in **Figure S1**. This protocol was designed with the same custom relaxation- and diffusion-weighted EPI sequence as the 45 min one, and comprised 333 unique (τ_E,b,b_Δ,Θ,Φ) data-points acquired with a repetition time of 2.5 s. The imaging field of view was set to 230×230×55 mm^3, with a voxel-size of 2.3×2.3×5 mm^3. Four different b-tensor “shapes” were implemented with the five distinct waveforms described in the *Methods* section of the main text: a set of four spectrally-tuned waveforms (used to acquire data at b_Δ = –0.5, 0, 0.5, and 1), and a standard non-tuned Stejskal-Tanner waveform yielding b_Δ = 1. Spherically encoded data (b_Δ = 0) was acquired in two sets: a first one of 28 points equally distributed over seven b-values (b = 0.2, 0.5, 0.8, 1.1, 1.4, 1.7, and $2 \cdot 10^9$ sm^{-2}), and a second set of 16 points equally distributed over 4 b-values (b = 0.2, 0.8, 1.4, and $2 \cdot 10^9$ sm^{-2}). The first set was repeated at three echo-times (τ_E = 80, 100 and 120 ms), while the second set was acquired at τ_E = 140 ms. All b_Δ = 0 points were measured with the same gradient waveform orientation. Linearly encoded (b_Δ = 1) data was measured across 64 directions spread out over four b-values (6, 12, 16, and 30 directions at, respectively, b = 0.2, 0.8, 1.4, and $2 \cdot 10^9$ sm^{-2}); 32 b_Δ = 0.5 points were distributed between three different b-values (6, 10, and 16 directions at, respectively, b = 0.2, 0.8, and $1.4 \cdot 10^9$ sm^{-2}); and b_Δ = –0.5 data was acquired over 32 directions distributed over three b-values (6, 10, and 16 directions at, respectively, b = 0.2, 1, and $2 \cdot 10^9$ sm^{-2}). Linear (b_Δ = 1) and planar (b_Δ = –0.5) encoded datasets were repeatedly acquired at two different echo-times (τ_E = 80, and 120 ms), while the b_Δ = 0.5 points were collected at τ_E = 80 ms. The various (b,b_Δ,Θ,Φ) sets were acquired using spectrally-tuned waveforms (Callaghan and Stepišnik, 1996). The Stejskal-Tanner waveform was used to measure 9 additional b_Δ = 1 data-points (3, and 6 directions at, respectively, b = 0.2, and $0.8 \cdot 10^9$ sm^{-2}) at an echo-time of τ_E = 50 ms. All b-tensor orientations were defined according to an electrostatic repulsion scheme (Bak and Nielsen, 1997;Jones et al., 1999).

The abbreviated dataset was inverted with the Monte Carlo algorithm described in the main document. The resulting 5D R_2-**D** distributions and parameter maps are compiled in **Figure S2-Figure S6**, which are direct reproductions of **Figures 2-6** of the main text. Overall, the analysis of the 15 min data provides the same results as the inversion of the 45 min dataset, with each figure in the Supplementary Material being comparable to its main text counterpart.

FIGURES

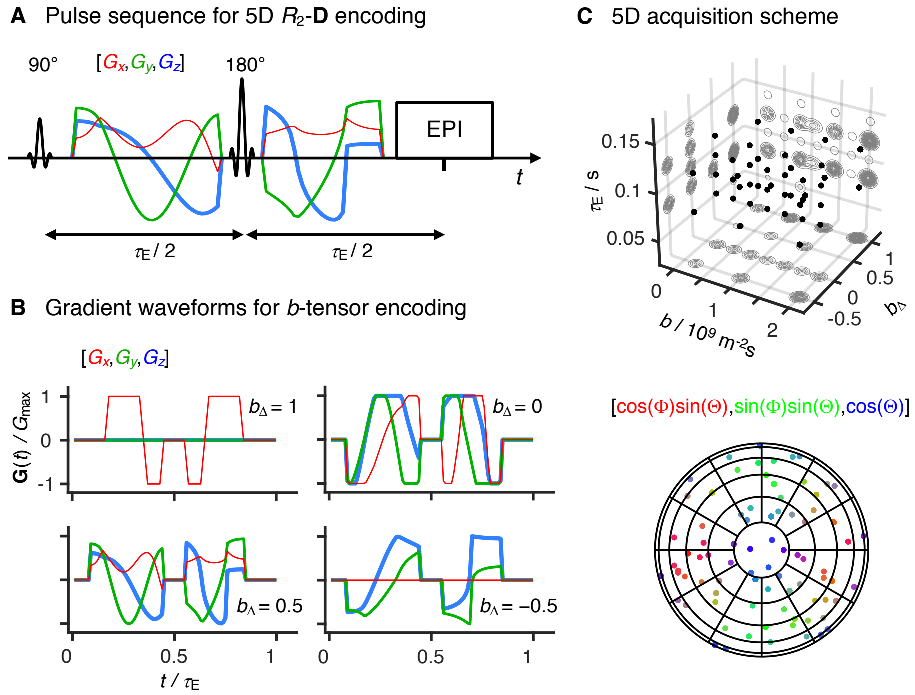

Figure S1. 15 min acquisition protocol for 5D relaxation-diffusion MRI. (A) Pulse sequence for acquiring images encoded for relaxation and diffusion in a 5D space defined by the echo time τ_E, and b-tensor trace b, normalized anisotropy b_Δ, and orientation (Θ,Φ). An EPI image readout block acquires the spin-echo produced by slice-selective 90° and 180° radio-frequency pulses. The 180° pulse is encased by a pair of gradient waveforms allowing for diffusion encoding according to principles from multidimensional solid-state NMR (Topgaard, 2017) (red, green, and blue lines). The signal is encoded for the transverse relaxation rate R_2 by varying the value of τ_E. (B) Numerically optimized gradient waveforms (Sjölund et al., 2015) yielding four distinct b-tensor shapes (b_Δ = –0.5, 0.0, 0.5, and 1) (Eriksson et al., 2015). (C) Acquisition space coordinates probed in this work. The τ_E, b, and b_Δ points are indicated in a 3D scatter-plot (top), complemented with contour lines representing 2D projections of the sampling density of the various coordinate points. The acquired (Θ,Φ) orientations are shown as discrete coordinates on an azimuthal projection of the unit sphere (bottom), color-coded according to the Cartesian components of the b-tensor orientation vector: [R,G,B] = [cosΦsinΘ, sinΦsinΘ, cosΘ].

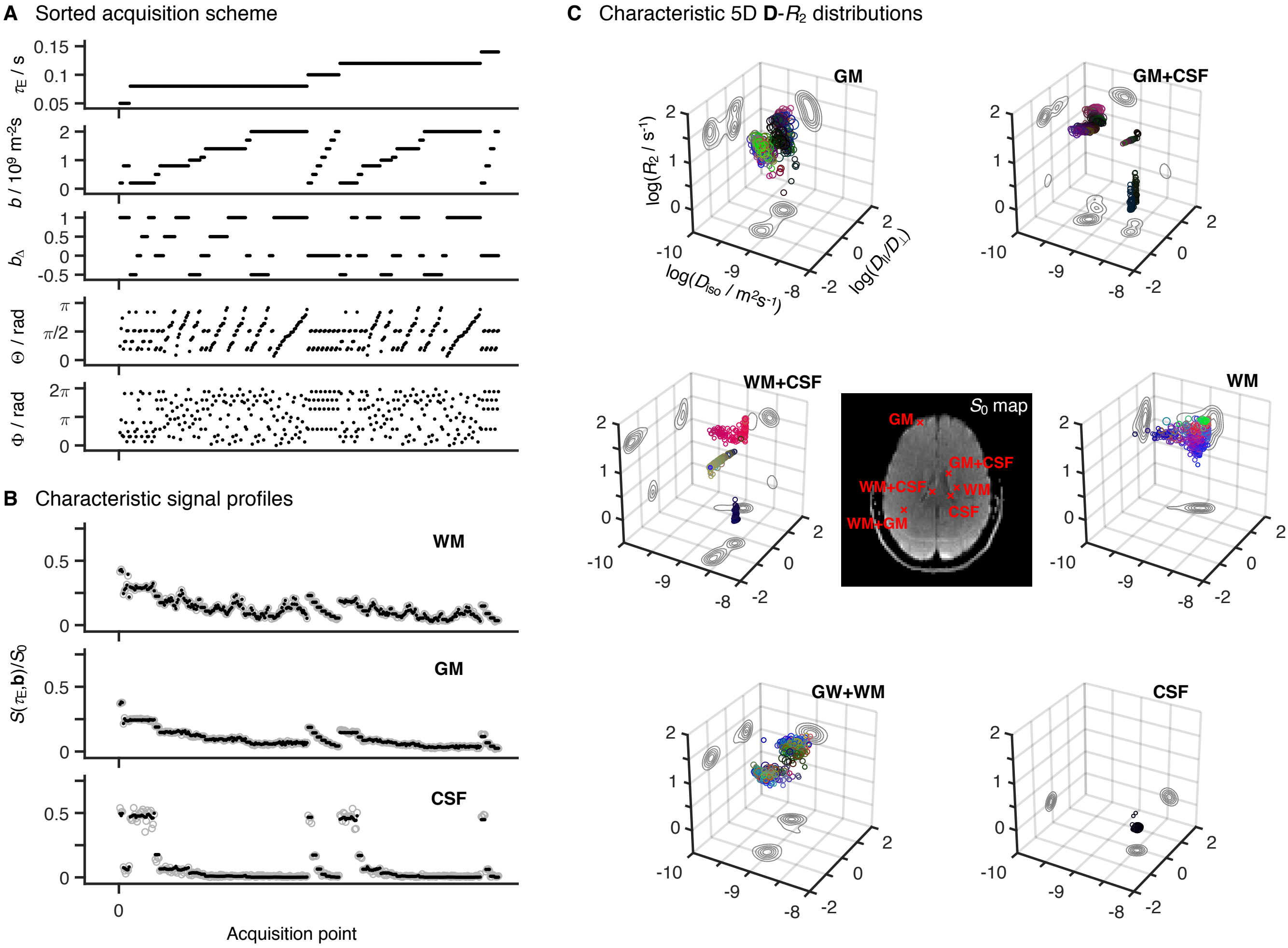

Figure S2. 15 min protocol – Representative 5D relaxation-diffusion encoded signals and distributions for selected voxels in a living human brain. (A) Acquisition scheme showing τ_E, b, b_Δ, Θ, and Φ as a function of acquisition point. (B) Experimental (gray circles) and fitted (black points) $S(\tau_E,\mathbf{b})$ signals from three representative voxels containing white matter (WM), gray matter (GM), and cerebrospinal fluid (CSF). The presented signal data was acquired according to the scheme shown in panel A and is drawn with the same horizontal axis. (C) Nonparametric R_2-$\mathbf{D}$ distributions obtained for both pure (WM, GM, CSF) and mixed (WM+GM, WM+CSF, GM+CSF) voxels. The discrete distributions are reported as scatter plots in a 3D space of the logarithms of the transverse relaxation rate R_2, isotropic diffusivity D_{iso}, and axial-radial diffusivity ratio $D_{||}/D_{\perp}$. The diffusion tensor orientation (θ,ϕ) is color-coded as [R,G,B] = $[\cos\phi \sin\theta, \sin\phi \sin\theta, \cos\theta] \cdot |D_{||}-D_{\perp}|/\max(D_{||}, D_{\perp})$ and the circle area is proportional to the statistical weight of the corresponding component. The contour lines on the sides of the plots represent projections of the 5D $P(R_2,\mathbf{D})$ distribution onto the respective 2D planes. Panels (B) and (C) display the signals $S(\tau_E,\mathbf{b})$ and corresponding $P(R_2,\mathbf{D})$, respectively, for the same WM, GM, and CSF voxels.

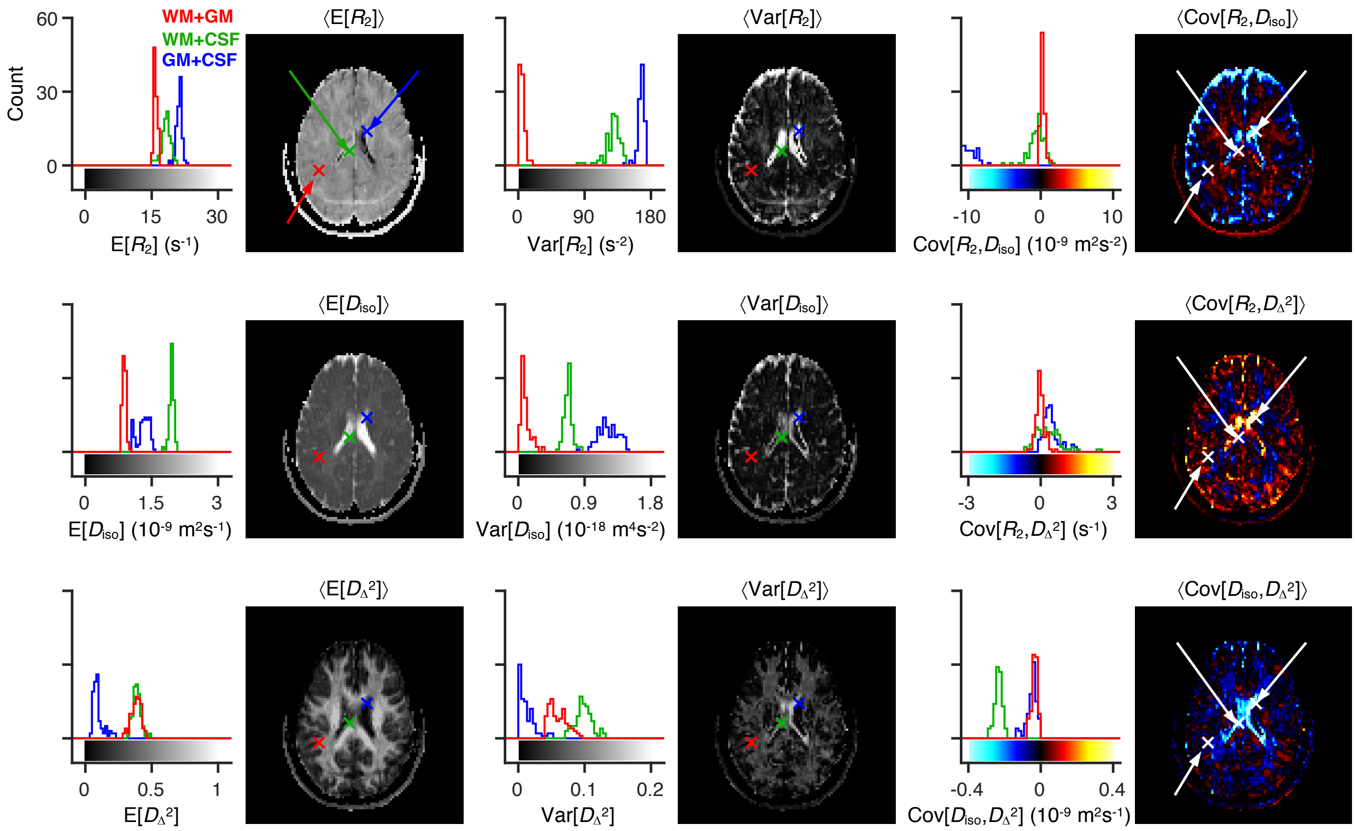

Figure S3. 15 min protocol – Statistical measures derived from the relaxation-diffusion distributions. The ensemble of 96 distinct $P(R_2,\mathbf{D})$ solutions was used to calculate averages E[x], variances Var[x] and covariances Cov[x,y] of all combinations of transverse relaxation rate R_2, isotropic diffusivity D_{iso}, and squared anisotropy $D_\Delta{}^2$. The statistical measures were all derived from the entire R_2-$\mathbf{D}$ distribution space on a voxel-by-voxel basis. Histograms are used to represent the parameter sets calculated for three voxels containing binary mixtures of white matter WM, gray matter GM, and cerebrospinal fluid CSF. Each histogram comprises 96 estimates of a single statistical measure. The averages of statistical measures, ⟨E[x]⟩, ⟨Var[x]⟩ and ⟨Cov[x,y]⟩, are displayed as parameter maps whose color scales are given by the bars along the abscissas of the histograms. The crosses and arrows identify the heterogeneous voxels analyzed in the histograms; notice that the signaled points correspond to the average (as measured by the median) of the ensembles of plausible solutions shown in the histograms.

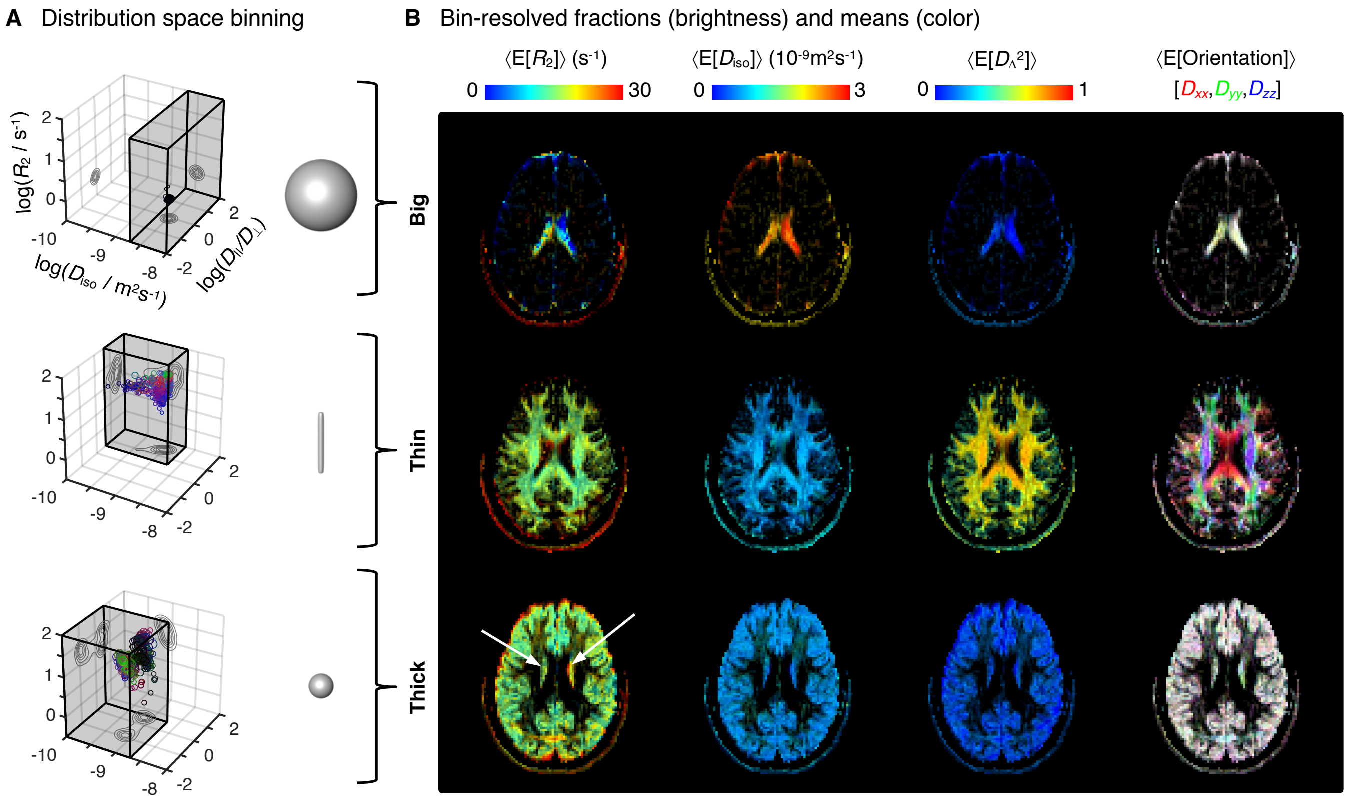

Figure S4. 15 min protocol – Parameter maps with bin-resolved means of the relaxation-diffusion distributions. (A) Division of the R_2-**D** distribution space into different bins. The distribution space was separated into three bins (gray volumes) named ‘Big’, ‘Thin’, and ‘Thick’ that loosely capture the diffusion features of cerebrospinal fluid CSF, white matter WM, and gray matter GM, respectively. The 3D scatter plots display the nonparametric R_2-**D** distributions corresponding to the CSF (top), WM (middle), and GM (bottom) voxels selected in **Figure S2**. Superquadratic tensor glyphs are used to illustrate the representative **D** captured by each bin. (B) Parameter maps of average per-bin means (color) of transverse relaxation rate ⟨E[R_2]⟩, isotropic diffusivity ⟨E[D_{iso}]⟩, squared anisotropy ⟨E[D_Δ^2]⟩, and diffusion tensor orientation ⟨E[Orientation]⟩. The orientation maps (column 4) are color-coded as [R,G,B] = [D_{xx},D_{yy},D_{zz}]/max(D_{xx},D_{yy},D_{zz}), where D_{ii} are the diagonal elements of laboratory-framed average diffusion tensors estimated from the various distribution bins. Brightness indicates the signal fractions corresponding to the ‘Big’ (row 1), ‘Thin’ (row 2), and ‘Thick’ (row 3) bins. The white arrows identify deep gray matter structures.

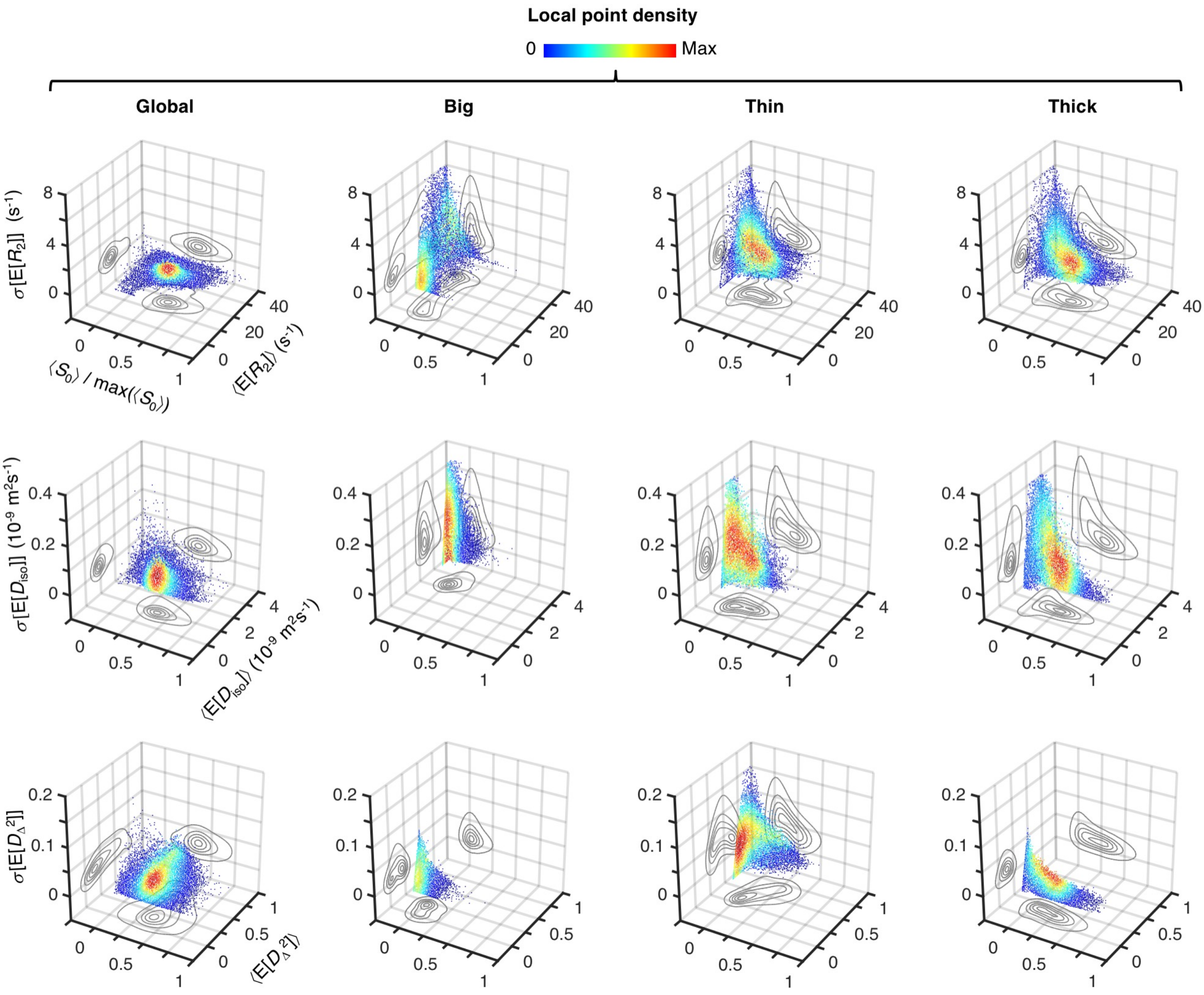

Figure S5. 15 min protocol – Uncertainty estimation of the statistical measures derived from the relaxation-diffusion distributions. 3D density (color) scatter plots showing the relationship between average initial signal intensity $\langle S_0 \rangle$, the average of mean values derived from the R_2-**D** distributions $\langle \mathrm{E}[x] \rangle$, and their corresponding uncertainties $\sigma[\mathrm{E}[x]]$. For display purposes, signal intensity values were normalized to the maximum recorded $\langle S_0 \rangle$, $\max(\langle S_0 \rangle)$. The contour lines on the side planes show 2D projections of the point density function defining the spatial distribution of data points. The average mean values of transverse relaxation rate $\langle \mathrm{E}[R_2] \rangle$ (row 1), isotropic diffusivity $\langle \mathrm{E}[D_{\mathrm{iso}}] \rangle$ (row 2), and squared anisotropy $\langle \mathrm{E}[D_\Delta^2] \rangle$ (row 3) were computed from all voxels whose $\langle S_0 \rangle$ was greater than 5% of $\max(\langle S_0 \rangle)$. The resulting dataset comprises 43754 voxels spread throughout all slices of the acquired 3D volume. The uncertainties of $\langle \mathrm{E}[R_2] \rangle$, $\langle \mathrm{E}[D_{\mathrm{iso}}] \rangle$, and $\langle \mathrm{E}[D_\Delta^2] \rangle$ correspond to the median absolute deviation between measures extracted from 96 independent solutions of Equation (2) from the main text: $\sigma[\mathrm{E}[R_2]]$, $\sigma[\mathrm{E}[D_{\mathrm{iso}}])$, and $\sigma[\mathrm{E}[D_\Delta^2]]$, respectively. All displayed data was derived from both the entire R_2-**D** space (column 1), and the 'Big' (column 2), 'Thin' (column 3), and 'Thick' (column 4) bins defined in **Figure S4A**.

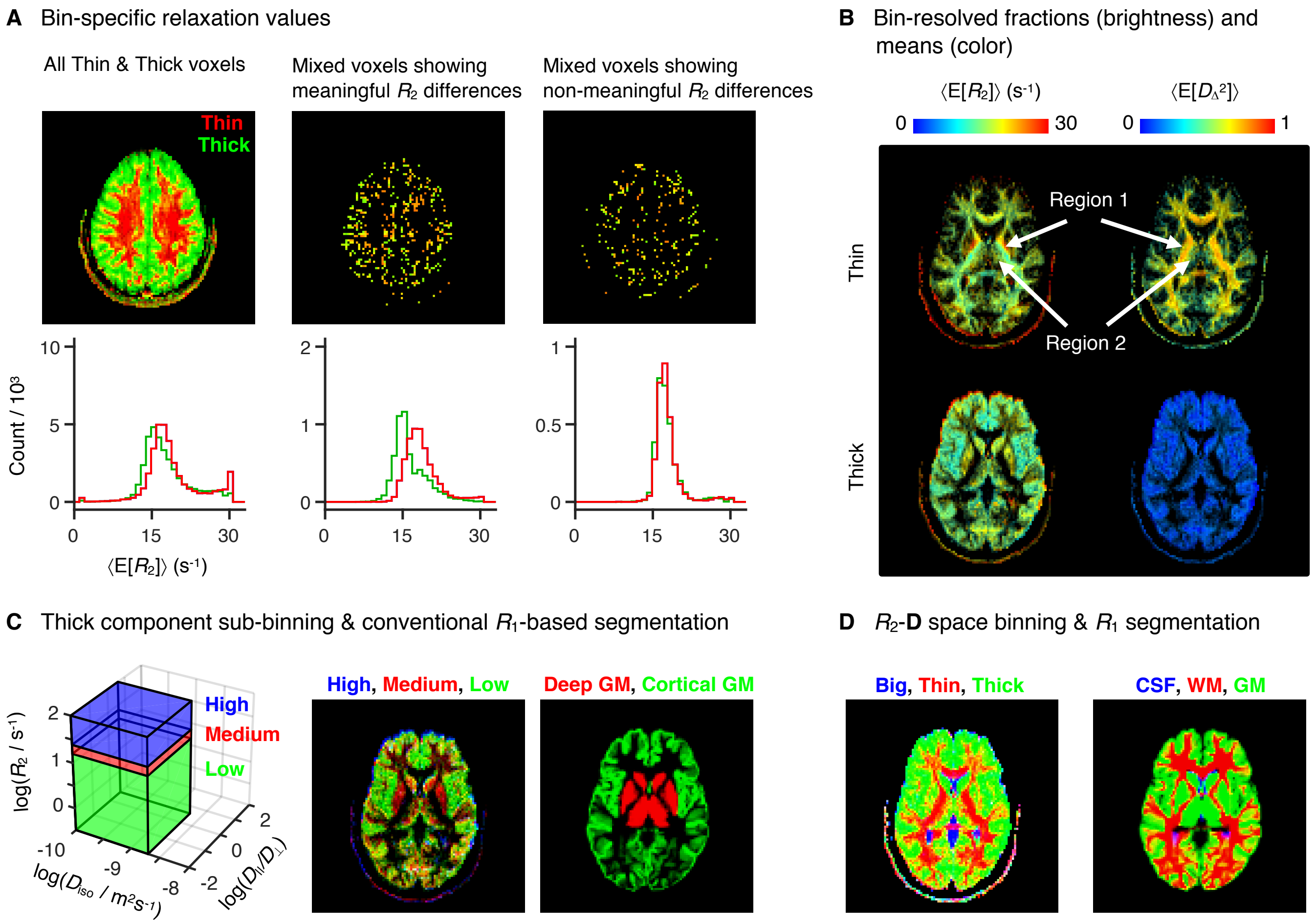

Figure S6. 15 minutes protocol – Per-bin relaxation properties and tissue composition. (A) Transverse relaxation properties specific to each of the 'Thin' (red) and 'Thick' (green) bins defined in **Figure S4A**. The color-coded composite images (top) and histograms (bottom) display the fractional populations and average mean transverse relaxation values $\langle E[R_2] \rangle$ of the two bins. The first column displays all of the 'Thin' and 'Thick' voxels, while the two other columns focus on 'Thin'+'Thick' mixtures wherein the bin-specific $\langle E[R_2] \rangle$ values exhibit either significant (second column) or non-significant (third column) differences. (B) Bin-resolved signal fractions (brightness) and average per-bin means (color) of R_2, and squared anisotropy D_Δ^2. Regions 1 and 2 identify the pallidum and the thalamus, respectively. (C) Subdivision of the 'Thick' bin into three different R_2 sub-spaces. The contributions from different sub-bins are compared with a high-resolution R_1-weighted image segmented into four different tissues: white matter WM, cortical gray matter GM, deep GM, and cerebrospinal fluid CSF. Additive color maps display the spatial distribution of sub-bin fractions (from low to high R_2: green, red, blue), and of cortical (green) and deep (red) GM. (D) Color-coded composite images showing the contributions of different bins (red=Thin, green=Thick, blue=Big) and conventional R_1-based segmentation labels (red=WM, green=cortical+deep GM, blue=CSF).

REFERENCES

Bak, M., and Nielsen, N. C.: REPULSION, a novel approach to efficient powder averaging in solid-state NMR, J. Magn. Reson., 125, 132-139, 10.1006/jmre.1996.1087, 1997.

Callaghan, P. T., and Stepišnik, J.: Generalized analysis of motion using magnetic field gradients, in: Advances in magnetic and optical resonance, Elsevier, 325-388, 1996.

Eriksson, S., Lasic, S., Nilsson, M., Westin, C. F., and Topgaard, D.: NMR diffusion-encoding with axial symmetry and variable anisotropy: Distinguishing between prolate and oblate microscopic diffusion tensors with unknown orientation distribution, J. Chem. Phys., 142, 104201, 10.1063/1.4913502, 2015.

Jones, D. K., Horsfield, M. A., and Simmons, A.: Optimal strategies for measuring diffusion in anisotropic systems by magnetic resonance imaging, Magn. Reson. Med., 42, 515-525, doi:10.1002/(SICI)1522-2594(199909)42:3<515::AID-MRM14>3.0.CO;2-Q, 1999.

Sjölund, J., Szczepankiewicz, F., Nilsson, M., Topgaard, D., Westin, C.-F., and Knutsson, H.: Constrained optimization of gradient waveforms for generalized diffusion encoding, J. Magn. Reson., 261, 157-168, 10.1016/j.jmr.2015.10.012, 2015.

Topgaard, D.: Multidimensional diffusion MRI, J. Magn. Reson., 275, 98-113, 10.1016/j.jmr.2016.12.007, 2017.
